# Production and characterization of starch nanoparticles by mild alkali hydrolysis and ultra-sonication process

**DOI:** 10.1038/s41598-020-60380-0

**Published:** 2020-02-26

**Authors:** Mudasir Ahmad, Adil Gani, Ifra Hassan, Qingrong Huang, Hassan Shabbir

**Affiliations:** 1 Department of Food Science and Technology, University of Kashmir, Srinagar, 190006 India; 2 Department of Food Science, Rutgers University, 65 Dudly Road, New Jersey, NJ 08901 USA

**Keywords:** Biochemistry, Nanoscience and technology

## Abstract

In this report, synthesis of the starch nanoparticles from underutilized and cheap sources viz: Horse chestnut (HS), Water chestnut (WS) and Lotus stem (LS) by using mild alkali hydrolysis and ultra-sonication process has been presented. The particles were characterized by Differential scanning colorimeter (DSC), X-Ray Diffraction (XRD), Rheology, Scanning electron microscopy (SEM) and Fourier transform infra-spectroscopy (ATR-FTIR). The particle size measurements, functional properties and antioxidant potential of starch nanoparticles were also analyzed. The experimental results revealed that the average particle size diameter of Horse chestnut starch nanoparticles (HSP), Water chestnut starch nanoparticles (WSP) and Lotus stem starch nanoparticles (LSP) was found to be 420, 606 and 535 nm, respectively. We observed a notable increase in the water absorption capacity but decreased capacity for oil absorption in the starch nano-particles. SEM images revealed damaged starch granules after size reduction. Additionally, loss of crystallinity and molecular order was observed from XRD and ATR-FTIR spectra. It was concluded that the starch nanoparticles have better thermal stability, increased viscosity and antioxidant properties.

## Introduction

Starch is a natural biopolymer, which is abundantly found in nature and also major component of our daily diet. It is mainly found in plant roots, staple crops and cereals such as rice, maize, wheat, barley, corn, tapioca, potato and others^1^. Starch is composed of linear chain molecule; amylose and branched chain molecule; amylopectin. These two starch components are assembled in the form of granules with the size ranging from 1 to 100 µm^2^. Starch has various applications, it has been used as thickening, gelling, stabilizing in a wide variety of foods and non-food products^3,4^. Starch is also used for drug and bioactive delivery systems^3,5,6^. The native form has however many limitations such as poor solubility, retro-gradation, limited digestibility and poor functional properties. For this reason, various physical, chemical and enzymatic methods have been employed to reduce these limitations or to add new attributes. Currently, starch nano particles are gaining more interest for improved quality and wide applications. They have been considered as the promising biomaterials for novel utilization in foods, cosmetics, and medicines as well as various composites^7^. There are various techniques for starch nano particle preparations including hydrolysis by acid, enzymes or combination of two, regeneration and mechanical treatments using extrusion, irradiation, ultrasound or precipitation by co solvent^3,8^. The easy and cost-effective methods for starch and starch derivative nanoparticles are always of paramount importance. Among various such methods, nano-precipation and ultra-sonication are very simple and reliable methods for nanoparticle production with desired size. The precipitation process involves a drop wise addition of a dilute starch solution into a non-solvent and ultra-sonication reduces the size by breaking the covalent bonds in polymeric material due to intense shear forces or mechanical effects associated with collapsing of micro bubbles by sound waves, it is simple, effective and environment friendly procedure^3,9,10^. Starch nanoparticles (SNP) were prepared by using ultrasound method without chemical additives from cassava, corn, yam and other sources of starch^3,11^. However the combination of physical and chemical processes produced nanoparticles with more desired properties^12^. So far starch from various sources like cereals, millets, tubers and others have been extracted and synthesized to nanoparticles^13–15^, but the novel and cheap sources are of great importance from commercial point of view. Therefore, we selected the extraction of starch from crops like horse chestnut, water chestnut and lotus stem, as they remain mostly underutilized at commercial level and huge quantum of produce generally goes waste. The starch nanoparticles were synthesized using novel methodology involving mild chemical and mechanical combinations of alkali hydrolysis and ultra-sonication. This technique is simple and convenient in terms of safety, cost and can give better yield with desired particle size. The morphological, rheological, thermal and functional properties of starch nanoparticles were investigated. The study also reported the anti-oxidant properties of starch nano-particles.

## Results and Discussions

### Particle size and zeta potential

The average particle size, zeta potential and particle size distribution of starch nano particles of HSP, LSP and WSP are shown in Table 1. The average particle size diameter of HSP, LSP and WSP was found to be 420.33 ± 20.21, 606.31 ± 15.32 and 535.21 ± 18.54 nm, having poly dispersity index of 0.456 ± 0.12, 0.798 ± 0.07 and 0.37 ± 0.09 with zeta potential of −25.38 ± 3.13, −15.3 ± 2.12 and −41.29 ± 3.23 mV respectively. The lowest particle size of HSP could be related to the difference in branching pattern of amylopectin within the starch. HS possess A type starch having unbranched amylopectin chains^15^ which could be easily broken down by ultra-sonication through a process called cavitation and therefore resulted in smaller size of the particles^16^. Furthermore, the zeta potential of all starch nano particles was found negative. Zeta potential predicts the long-term stability of the nanoparticle. The WSP displayed significantly higher negative zeta potential (−41.29 ± 3.23 mV) making them more stable than HSP and LSP. The high zeta potential increases the electrostatic repulsions between the particles, which results in the decrease of Van der Waals forces of attraction. The Van der Waals forces are responsible for particle agglomeration that eventually leads to bigger particles^17^.

**Table 1:** Particle size, Zeta potential and Polydispersity index of starch nanoparticles.

Sample	Hydrodynamic diameter (nm)	Polydespersity index	Zeta Potential (mV)
HSP	420.33 ± 20.21^c^	0.456 ± 0.12^b^	−25.38 ± 3.13^b^
LSP	606.31 ± 15.32^a^	0.798 ± 0.07^a^	−15.3 ± 2.12^c^
WSP	535.21 ± 18.54^b^	0.37 ± 0.09^c^	−41.29 ± 3.23^a^

HSP, WSP and LSP represent starch nanoparticles from Horse chestnut, Water chestnut and Lotus stem, respectively. ^a,b,c^The different small superscript letters on the average data with standard deviation (±) in the same column are significantly different (p < 0.05).

### Water and oil absorption capacity

**Figure 1:**
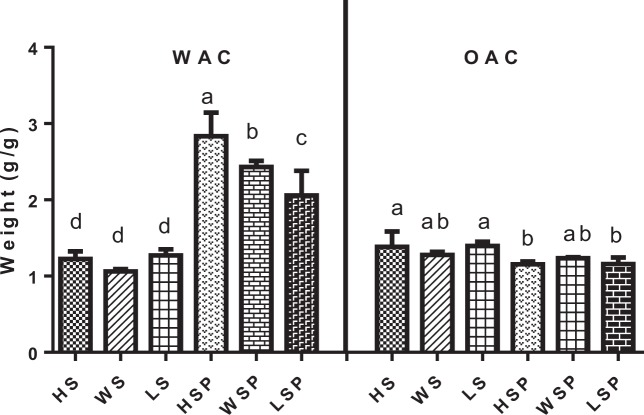
Functional properties of native and nano particles of starch. Footnote: Data with different superscript above the bars are significantly different (p < 0.05). HSP, WSP and LSP represent starch nanoparticles while as HS, WS and LS represent native starch from Horse chestnut, Water chestnut and Lotus stem, respectively. WAC (water absorption capacity); OAC (Oil absorption capacity).

The water and oil absorption capacity of starch and starch nanoparticles are depicted in Fig. 1. The water absorption capacity (WAC) of starch nanoparticles was found to be higher than native starch. Water absorption capacity is a function of water holding ability of the starch sample. It is an important processing parameter that has implication for viscosity. WAC of starch nano-particles was seen in concentration ranging from 2.05 ± 0.32–2.83 ± 0.311 g/g, whereas native starch showed 1.06 ± 0.03–1.27 ± 0.08 g/g WAC. The significant increase in water absorption capacity can be related to an increase in the surface area of starch nanoparticles. The combined effect of alkali and sonication may lead to reduced or broken amylopectin crystalline part, which has less affinity towards water molecule. The breakage of inter molecular bonds by starch hydrolysis allowed the hydrogen bonding sites to engage more water in starch nanoparticles. On contrary, the Oil absorption capacity of starch decreased significantly after size reduction. The OAC in native and nano sized starch was investigated in the concentration ranging 1.27 ± 0.05–1.38 ± 0.2 and 1.15 ± 0.18–1.23 ± 0.11 g/g respectively. Several studies have also shown OAC/WAC in the similar range^18^. The OAC is associated with hydrophobic inner surface of loosely helical chains of amylose that is not able to hold water well, thus lipids or oils replaces them easily and the extent of hydrophobic channel depends on the size of intra helical included molecules^19^. The decrease in OAC can therefore be correlated with reduction in size and change in the polymeric intermolecular network influencing the hydrophobicity of starch nanoparticles.

Data with different superscript above the bars are significantly different (p < 0.05).HSP, WSP and LSP represent starch nanoparticles while as HS, WS and LS represent native starch from Horse chestnut, Water chestnut and Lotus stem, respectively. WAC (water absorption capacity); OAC (Oil absorption capacity).

### Structure elucidation of starch nanoparticles by ATR- FTIR (Attenuated Total Reflectance Fourier Transform Infrared) Spectroscopy

**Figure 2:**
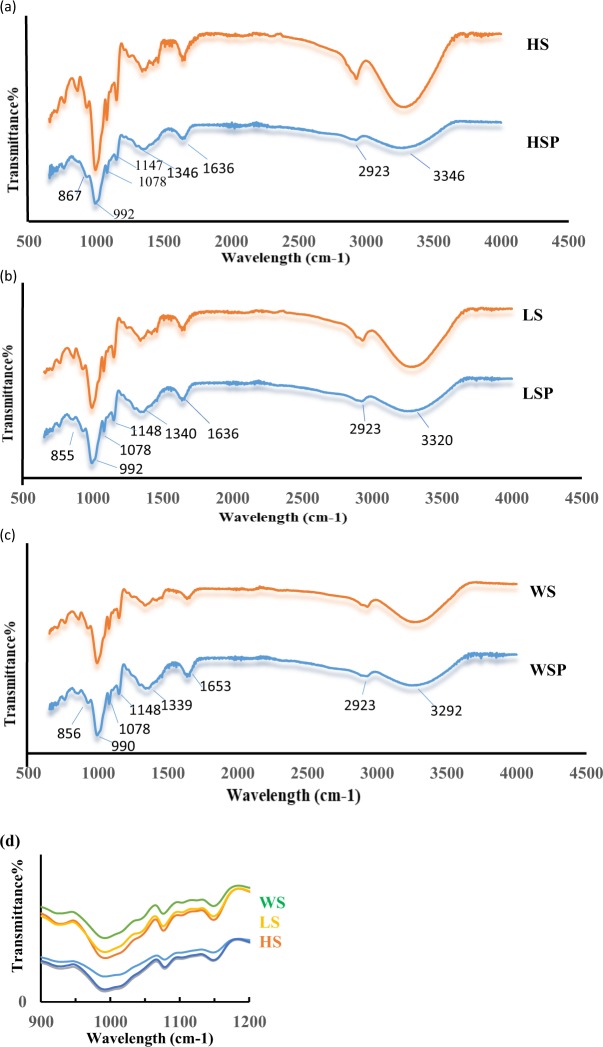
(**a–c**) ATR- FTIR curves of native and nano starch particles. Footnote: HSP, WSP and LSP represent starch nanoparticles while as HS, WS and LS represent native starch from Horse chestnut, Water chestnut and Lotus stem, respectively.

Figure 2(a–c) shows the FTIR spectra of native starch and nano starch particles. The FTIR spectra depicts almost identical characteristic bands with slight increase/decrease in the intensity of the peaks of HSP, LSP and WSP in comparison to peaks of native Starch (HS, LS and WS). The FTIR spectra showed the strong absorption peak between 3290–3246 cm^−1^ which is attributed to the -OH stretching^20^ and its breadth indicated the extent of formation of inter- and intra-molecular hydrogen. In starch nanoparticles, the peaks of O–H stretching shifted to higher wavelength for WSP, LSP and HSP. This could be attributed to the loss of the crystalline structure and exposure of -OH groups of the starch molecule due to alkalization and sonication process^21,22^. The other characteristic bands were observed at wavenumbers of around 2923 cm^−1^ assigned to -CH_2_ stretching vibrational modes bands and 1147, 1078 and 990 cm^−1^ associated with the stretching vibration of the C-O bond, C-O-H and C-O-C groups in the anhydrous glucose ring, respectively^23^. The characteristic peak at 1643 cm^−1^ is due to the presence of bounded water in starch and it does not show any evident change in the peak intensity after size reduction of starch. The characteristic peaks at 855, 856 and 867 cm^−1^ for LSP, WSP and HSP respectively, indicate the existence of β-glycosidic bonds in the samples^6,24^.

FTIR spectroscopy was also used to determine the crystallinity of starch by characterizing the changes that occur in the semi crystalline and amorphous domains within starch granules. The IR band between 950–1050 cm^−1^ with high peak intensity at 995 cm^−1^ possess shoulder at the wavenumber of 1018 cm^−1^ and 1047 cm^−1^ **(**Fig. 2d) indicating amorphous character and crystalline order of starch^25^. The ratio of absorbance of the bands at 1047/1018 cm^−1^ and 995/1018 cm^−1^ was quantified to obtain the degree of order of starch^25,26^ as shown in Table 2. The ratio of absorbance band at 1047/1018 cm^−1^ and 995/1018 cm^−1^ decreased after size reduction of starch indicating decrease in crystalline structure of starch and generation of its amorphous phase. Thus, it is clear from the infrared spectra that alkali modification and ultra-sonication of starches is accompanied by some changes in the physico-chemical structure of starch.

**Table 2:** Relative crystallinity and molecular order of native and nanostarch particles quantified by ATR-FTIR.

Sample	1047/1018* (cm^−1^)	995/1018* (cm^−1^)
HSP	0.638	1.075
HS	0.685	1.192
LSP	0.634	1.104
LS	0.689	1.206
WSP	0.634	1.110
WS	0.678	1.195

HSP, WSP and LSP represent starch nanoparticles from Horse chestnut, Water chestnut and Lotus stem, respectively.

*Ratio of absorbance at wavenumber 1047/1018 cm^−1^ and 995/1018 cm^−1^.

### Thermal characterization by differential scanning calorimeter (DSC)

**Figure 3:**
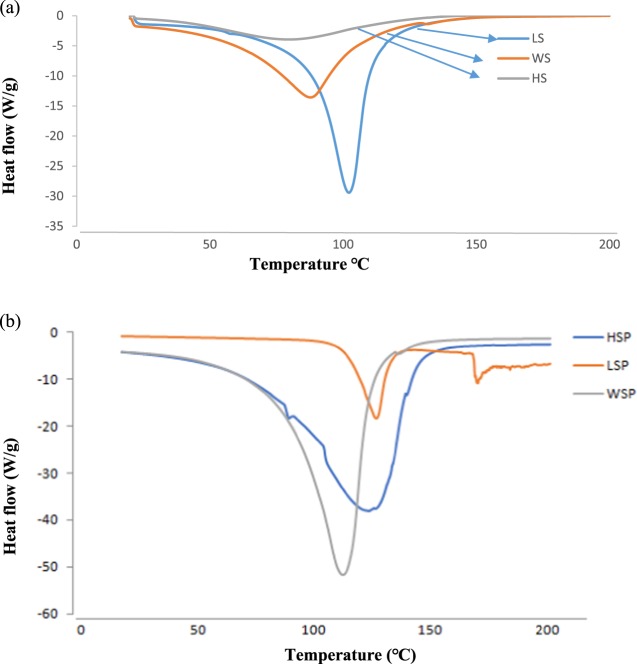
(**a,b**) Thermal analysis by Differential Scanning Colorimeter (DSC). Footnote: HSP, WSP and LSP represent starch nanoparticles while as HS, WS and LS represent native starch from Horse chestnut, Water chestnut and Lotus stem, respectively.

The thermal characteristics of native starch and starch nano-particles have been performed by DSC. The obtained values for each of the samples have been specified in Fig. 3(a,b). All the samples showed different initial temperatures (T_0_) and final temperatures (T_C_). The peaks for WS, LS, HS, WSP, LSP and HSP have been recorded between 49.26–124.23 °C, 69.88–125.74 °C, 50.76–119.35 °C, 52.76–134.15 °C, 108.10–148.79 °C, and 51.99–144.61 °C respectively. The peak transition temperatures have been detected at 87.68 °C, 102.36 °C, 83.72 °C, 110.23 °C, 135.80 °C, and 121.81 °C for WS, LS, HS, WSP, LSP and HSP respectively. WSP showed a sharp peak at 135.80 °C corresponding to its melting point and its crystal structure. HSP starch curves displayed broad endotherm glass transition at vicinities 95 °C and a sharp endotherm melting transition has been shown by LSP at 160 °C. The broad glass transition could be ascribed to amorphous structure of starch in addition to endothermic events overlapping at gelation point of amylopectin^27^ WS, LS and HS showed lower transition temperature which could be attributed to the lower gelatinization enthalpy in native starches having values of −522, −685, −104 J/Kg respectively for WS, LS and HS. Ultra sonication of starch granules results in a serious disruption of the crystalline structure of clustered amylopectin and increase of hydrocarbon chain that can be related to an increase in Van der Waals and hydrogen bonds with greater stability and higher melting temperature apparently leading to nano-particles with low crystallinity or an amorphous character. Peak of the crystallization temperature was not recognized indicating that these nanoparticles have higher melting point and more amorphous structure. Similar results were reported by Hsanvand & Fathi^28^ in starch nanoparticles

### XRD analysis

**Figure 4:**
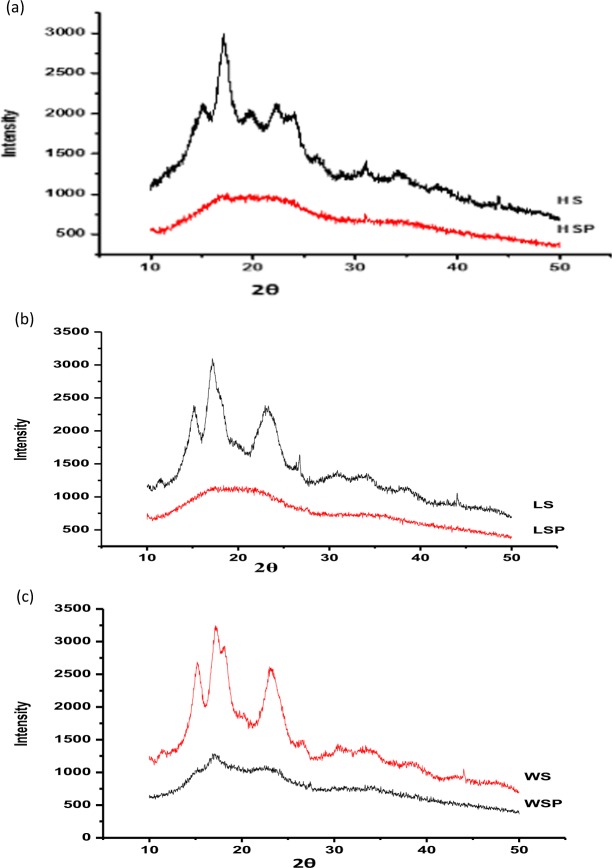
(**a–c**) Crystallographic structure of starch nanoparticles. Footnote: HSP, WSP and LSP represent starch nanoparticles while as HS, WS and LS represent native starch from Horse chestnut, Water chestnut and Lotus stem, respectively.

Crystal structures of synthesized starch nano-particles have been determined by X-Ray Diffraction (XRD) analysis as shown in Fig. 4(a–c). The crystallographic structure of HS, WS and LS have been already conducted in our previous study^3^, the obtained results and graphs were compared in this study with the nanoparticles of starch prepared from same source. The area under amorphous region and diffraction peaks has been calculated according to our previous method^3^ using Gaussian profile fits. XRD patterns revealed that the relative crystallinity (RC) of starch nano-particles decreased to 1.33, 5.56 and 14.46 in HSP, WSP and LSP, respectively. However, in the native starch the RC was found to be 13, 26 and 22% in HS, WS and LS respectively. The decrease in relative crystallinity can be related to an increase in amorphous region of the starch after size reduction. Nano sized starch showed either narrow or diminished diffraction peaks. The sharp or narrow peaks indicate large crystallite size. The crystallite size of the starch nanoparticles was estimated and found up to 3–10 nm. This also indicates that major peaks of diffraction do not exist and whole structure appeared like amorphous hump. Similar results were obtained from our previous studies where there is decrease in crystallite size of starch after sonication and alkalization of starch^3^. Methods like gamma irradiation, heat moisture treatment, microwave degradation and ultrasonic degradation have also shown decrease in crystallinity and crystallite size of starch^3,29–32^.

### Rheological properties

**Figure 5:**
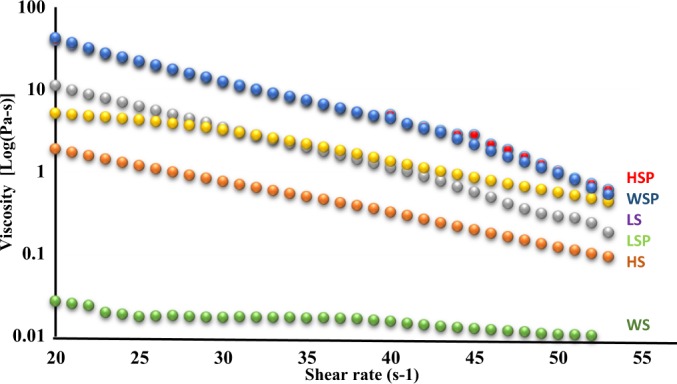
Viscosity of native and nano starch particles against shear rate. Footnote: HSP, WSP and LSP represent starch nanoparticles while as HS, WS and LS represent native starch from Horse chestnut, Water chestnut and Lotus stem, respectively.

Results for the rheological properties of native starch and starch nanoparticles are given in Fig. 5. The storage modulus G^/^ and Loss modulus G^//^ of starch was plotted against angular frequency (s^−1^). The G^/^ and G^//^ of starch nanoparticles was higher than the values obtained for native starch. Further, the difference between G^/^ and G^//^of starch nanoparticles was observed higher than native starch samples (Data not shown) suggesting the more elastic behavior of starch nanoparticle suspensions than native starch. The results for frequency sweep tests were also in agreement with the flow rate test conducted against the shear rate ranged from 0–60 s^−1^. The graph shown in Fig. 5 clearly indicates the higher viscosity in starch nanoparticle suspensions than native starch suspensions. However, with the increase in shear rate, the viscosity shows the slow decline indicating increase in liquidity. Several studies have also proven higher viscosity of starch nanoparticle suspensions than native starch^33,34^. The rheological properties of the starch are influenced by shape, size and distribution of the granules and also by the amylose content and granule–granule interaction^35^. According to obtained data, our study concluded with the results that decrease in size of starch at nano level increase its viscosity.

### Scanning electron microscopy (SEM)

**Figure 6:**
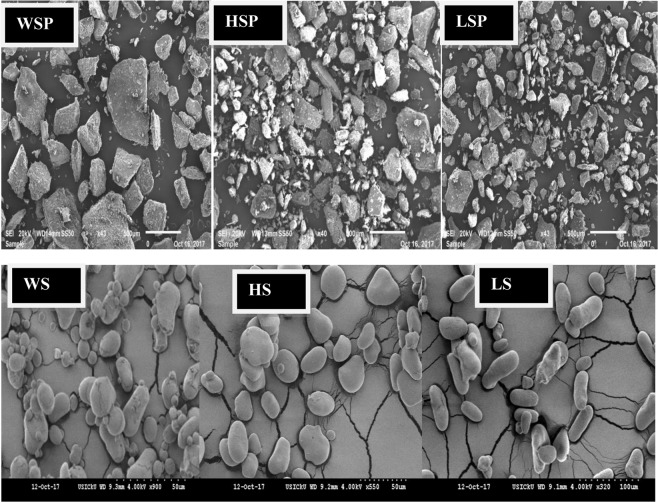
Micrographs of native and nano starch particles. Footnote: HSP, WSP and LSP represent starch nanoparticles while as HS, WS and LS represent native starch from Horse chestnut, Water chestnut and Lotus stem, respectively.

Scanning electron microscopy was used to observe the morphological analysis of the starch nano-particles including shape, size and porosity. The micrographs of native starch were also mentioned in our previous study. As shown in Fig. 6, the SEM micrographs of HS, LS, WS, HSP, LSP and WSP reveal the changes in starch that occur during synthesis of starch nanoparticles by ultra-sonication and alkali treatment. In contrary to nano-particles formed, native HS and WS starch granules are polygonal with a smooth surface, about 2–5 µm in size. Combined treatment of NaOH and ultra-sonication was suggested to play a significant role in a major change in the starch nanoparticles resulting in surface erosion, with notch and groove formation, breaking the cell walls to generate the smaller fragment and smaller sphere^36^. The granular structure of the native starch particles was smooth and round or oval shaped as was also reported in our previous studies^3,5,6,18^. The micrographs revealed that starch nano-particle granules are irregular in shape, size with no uniform pattern, which has been ascribed to the cavitation phenomenon inducing very high pressures and shear forces responsible for degradation of the external crystalline and amorphous layers of starch granules and therefore small segments can be produced. The surface appearance change was also observed in oat starch nano-particles by Falsafi *et al*.^37^. HSP and LSP showed uneven exterior with voids and their granular structure was found to be disrupted to large extent. LSP had rough surface with many cavities and the surface of WSP was relatively smooth showing few large fragments besides the presence of small particles within granules.

### Antioxidant properties

**Figure 7:**
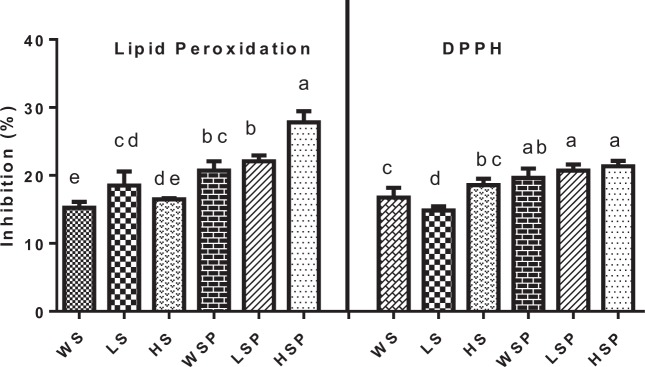
Antioxidant analysis of native and nano starch particles. Footnote: HSP, WSP and LSP represent starch nanoparticles while as HS, WS and LS represent native starch from Horse chestnut, Water chestnut and Lotus stem, respectively. Data with different superscript above the bars are significantly different (p < 0.05).

DPPH• scavenging activity of WS, LS, HS, WSP, LSP and HSP are shown in Fig. 7. DPPH radical scavenging potential was reported as: 16.74 ± 1.4%, 14.86 ± 0.58%, 18.58 ± 0.94%, 19.65 ± 1.35%, 20.72 ± 0.89% and 21.34 ± 0.80% for WS, LS, HS, WSP, LSP and HSP respectively, at a concentration of 20 mg/ml in DMSO. The antioxidant activity of starch was marginally increased after size reduction. The increase in DPPH activity could be due to chemical modification (alkali treatment) and ultra-sonication. Besides, formation of starch nano particles from novel methods like ultra-sonication and alkali hydrolysis enhances hydroxyl, superoxide anion radical scavenging capacity, chelating iron ion ability, reducing power and introduction of substitution groups (sulfate groups) into polysaccharide molecules that has been regarded to increases the hydrogen bonding ability of polysaccharides^38^. Moreover, it is known from studies that low molecular weight and monosaccharide composition can contribute to the antioxidant activity of polysaccharides^39^. Formation of nano-particles is accompanied with a decrease of molecular weight, hence improving the antioxidant potential. It is reported that ultrasonic treatment reduces the molecular weight and viscosity of starch solution^40^. Starch from some sources contain flavonoids and several studies have shown that flavonoids possess significant superoxide radical scavenging activity^41,42^. Superoxide anions have been observed to directly initiate lipid peroxidation, thus leading to oxidative damage in proteins, lipids and DNA. Lipid peroxidation was reported in the following trend: WS (15.26 ± 0.85%), LS (18.51 ± 2.08%), HS (16.48 ± 0.16%), WSP (20.70 ± 1.38%), LSP (22.09 ± 0.85%) and HSP (27.80 ± 1.65%).The results obtained were statistically significant (P < 0.05). WS, LS and HS inhibited lipid peroxidation at low extent (Fig. 7) while as WSP, LSP and HSP showed higher inhibitory effects on lipid peroxidation. Similar results were reported in sugiol isolated from *Metasequoia glyptostroboides*^43^.

## Conclusions

In this work, starch nano-particles from HS, WS and LS have been successfully synthesized by using a new technique involving mild treatment combinations of alkali hydrolysis and ultra-sonication. The starch nanoparticles were found to have better thermal stability with increased viscosity and good antioxidant properties. It was also determined from the obtained data that the starch nanoparticles were more amorphous having increased water absorption and decreased oil absorption capacity. These properties could be useful in particular food and drug formulations which may increase the commercial scope of HS, WS and LS in both food and pharmaceutical industries. Besides, it was suggested that the method of nanoparticle preparation was simple and convenient in terms of safety, cost and giving better yield with desired particle size.

## Experimental

### Materials and methods

All the analytical chemicals have been purchased from Sigma Aldrich (USA) and Himedia Laboratories Pvt Ltd. WS and LS was purchased from the local market of Srinagar, India, while HS were collected from the trees located in University of Kashmir, Srinagar, India at their maturity stage. The samples have been cleaned, de-hulled and then stored at 5 °C.

### Starch extraction

The samples of HS, WS and LS were cut into small pieces after de-hulling/peeling and then grounded along with some quantity of water in a domestic blender. The starch has been extracted using the alkaline steeping method^3^.

### Synthesis of starch nano-particles

The synthesis of starch nano-particles has been achieved by ultra-sonication and mild alkali hydrolysis. Starch solutions (1.5%) was preheated at 80 °C in 0.1M NaOH solution and continuously stirred for 30 min using magnetic stirrer. The starch slurry obtained was then sonicated at 40 KHz using the fixed probe sonicator (Q55-Qsonica, USA). The sonication time of 30 minutes was carried out at intervals of 5 minutes to avoid excessive heating. The resulting solution was then co-precipitated by ethanol in the ratio of 1:2 by drop wise addition of slurry to ethanol under continuous magnetic stirring. The precipitate was then collected by centrifugation of solution at 8000 rpm for 15 minutes and then lyophilized using freeze dryer (Telstar-Cryodos, Spain). The powdered samples were stored at 4 °C for further analysis.

### Zeta potential and particle size analysis of the starch nano-particles

In order to characterize particle suspension, particle size and zeta potential of starch nanoparticles is determined using particle size analyzer (MCR 102; Antonpar and Nano S Malvern Instruments). The 0.01% of sample has been dispersed in millo-Q water and sonicated for 30 minutes at 40 KHz in sonicator bath before particle size measurement. For measurement of zeta potential the 0.01% of sample was suspended in 0.1 mM KCl with pH adjusted to 6 and the samples have been equilibrated overnight before measurement.

### Water and oil absorption capacity (WAC/OAC)

One gram of each starch sample was mixed with 10 mL distilled water or vegetable oil and continuously stirred for 30 min at room temperature. The slurry was then centrifuged (5810 R, Eppendorf, Hamburg, Germany) for 15 minutes at 5000 rpm. The formed supernatant was decanted, and water/Oil absorption capacity was expressed as ratio of initial weight of sample to final weight of sample after oil/water absorption.

### Structure elucidation of starch nano-particles by ATR- FTIR (Attenuated Total Reflectance-Fourier Transform Infrared) Spectroscopy

The ATR-FTIR spectra of samples have been recorded on an Agilent FTIR-Cary 630 at room temperature at the wavelength region between 4000 and 650 cm^−1^. The FTIR spectra was examined by using Agilent Pro resolution software.

### Thermal characterization by differential scanning calorimeter (DSC)

The thermal behavior of samples was studied using DSC (Star^e^ systems, Mettler Toledo). The samples (5 mg) were placed in aluminum pans and 5 µl of milli Q water was added and mixed uniformly with the sample. The pan was hermetically sealed and heated from 20 °C to 200 °C at the rate of 10 °C/min. Characteristic temperatures of transitions were defined as T_o_(onset), T_p_ (peak of gelatinization), T_c_ (conclusion) and enthalpy of gelatinization (ΔH) was recorded.

### X-ray diffraction (XRD) analysis

The crystallographic structure has been analyzed by X-ray diffractometer (Shimadzu Lab X–XRD–6100). The operating conditions were X-ray line (λ = 1.5418 Å), voltage 40 kV and current 30 mA. The samples were loaded on aluminum plate and X-ray diffractions recorded from 10° to 50° for 2θ with scanning rate of 0.02/min. The relative crystallinity (RC) of starch was calculated as described by Rabek^44^ by the equation:

$$RC\, \% =Ac/(Ac+Aa)\times 100$$

where Ac is the crystalline area; Aa is the amorphous area on the X-ray diffractogram.

The crystallite size of the starch nano-particles has been calculated using Scherrer equation as given below:

1 $${\rm{D}}=\frac{{\rm{K}}{\rm{\lambda }}\,}{{\rm{\beta }}\,\cos \,{\rm{\theta }}}$$

where, D = mean crystallite size, λ = wavelength of the Cu_α_ X-ray line, K = shape factor (=0.9), β = full width of the diffraction peak at half maximum, and θ = Bragg’s diffraction.

### Rheology of starch nano-particles

The 6% starch nano-particles in water was heated to 90 °C for 20 minutes with constant gentle stirring till the gel was formed. The gel was allowed to cool at room temperature and rheological measurements were conducted using rheometer (Anton par, MCR 102) at 25 ± 0.1 °C on parallel plate geometry (CP50-1; diameter: 50 mm). The flow properties were determined with increasing shear rate, from 0.1 to 100 s and the delay time was 10 s. The viscoelastic range was determined through stress sweep tests at constant frequency of 1 Hz with a logarithmic increase of shear stress from 0.01 to 100 Pa.

### Scanning electron microscopy (SEM)

Microstructure of starch nano-particles have been monitored by scanning electron microscope (SEM, S-3000H, Hitachi, Japan). The samples were gold plated and different micrographs of samples were recorded.

### Antioxidant analysis

#### Sample preparation

The starch samples (20 mg/ml) was mixed with dimethyl sulphoxide (DMSO) and heated continuously till complete dissolution.

#### DPPH activity (1,1-dihpenyl-2-picrylhydrazyl)

The scavenging activity of 1,1-dihpenyl-2-picrylhydrazyl(DPPH) radical was measured according to the method described by Ahmad *et al*.^9^ with modifications. Briefly, 200 µL of each sample was added to 200 µL of a 0.01% of DPPH solution dissolved in dimethyl sulphoxide (DMSO). The final volume was adjusted to 3 ml by adding 2.6 ml of DMSO and incubated at 37 °C for 30 min. After incubation, absorbance was measured at 517 nm against a control (without sample). Percentage inhibition was calculated by using the formula:

2 $$ \% \,{\rm{Inhibition}}=[1-({\rm{Asample}}\,517/{\rm{Acontrol}}\,517)]\times 100$$

#### Lipid peroxidation inhibition

To measure the lipid peroxidation inhibition activity of samples, 100 µl of samples were added to1 mL of 0.2% linoleic acid, 0.2 mL of H_2_O_2_ (30 mM), 0.2 mL of ascorbic acid (100 mM) and 0.2 mL of ferric nitrate (20 mM). After this, the mixture was incubated at 37 °C in water bath for 1 h. The reaction was stopped by the addition of 1.0 mL TCA (trichloroacetic acid, 10% w/v), following with 1.0 mL of TBA (thiobarbituric acid, 1% w/v) and then tubes were placed in a boiling water bath for 20 min, which is followed by centrifugation at 5000 rpm for 15 minutes. The clear upper layer was collected and its absorbance was measure at 535 nm. Percentage inhibition was calculated by using equation.

3 $$ \% \,{\rm{Inhibition}}=[1-({{\rm{Asample}}}_{535}/{{\rm{Acontrol}}}_{535})]\times 100$$

#### Statistical analysis

All the analytical experiments have been carried in triplicate and statistical analysis was performed using commercial statistical package SPSS.10.1 (USA) and the data were assessed by analysis of variance (ANOVA) using Duncan’s multiple range test at 5% significance level. The results are presented as means ± standard deviation (n = 3).
